# Healthcare seeking among Swedish patients in opioid substitution treatment – a mixed methods study on barriers and facilitators

**DOI:** 10.1186/s13011-022-00434-w

**Published:** 2022-02-05

**Authors:** Katja Troberg, Karin Lundqvist, Helena Hansson, Anders Håkansson, Disa Dahlman

**Affiliations:** 1 Faculty of Medicine, Department of Clinical Sciences Lund, Psychiatry, Lund University, Lund, Sweden; 2 Malmö Addiction Centre, Skåne University Hospital, Malmö, Sweden; 3 Center for Primary Health Care Research, Department of Clinical Sciences, Lund University/Region Skåne, Malmö, Sweden; 4 School of Social Work, Faculty of Social Sciences, Lund University, Lund, Sweden

**Keywords:** Opioid substitution treatment, Healthcare seeking, Unmet healthcare needs, Barriers and facilitators, Stigma

## Abstract

**Background:**

Patients in opioid substitution treatment (OST) have poorer health than the general population. Thus, they do not seek somatic health care to the extent that is medically motivated. Barriers hindering patients from seeking medical help through the conventional healthcare system result in a high degree of unmet healthcare needs. Barriers to, and facilitators of, OST patients’ healthcare seeking have been sparsely examined.

**Methods:**

Mixed methods were employed. The quantitative part consisted of a cross-sectional questionnaire covering questions on physical health, healthcare seeking, and barriers thereof, which was collected from 209 patients in OST. A sub-sample of eleven OST patients participated in semi-structured interviews, for the qualitative part of the study, covering experience of healthcare, lifestyle, and self-images, expectations, and ideals of Swedish healthcare.

**Results:**

Confirmed by qualitative data, quantitative data revealed deprioritization, fear of stigma and of being treated badly, and problems in navigation throughout the healthcare system, leading to unsuccessful establishment of contact, being most common reasons for not seeking somatic healthcare. Thus, interviewees provided a deeper knowledge of the barriers stigma, lack of means to prioritize health and difficulties navigating throughout the healthcare system, leading to resignation and deprioritization. On-site primary healthcare (PHC) seemed to contribute to increased access and utilization of healthcare.

**Conclusion:**

Individual and structural barriers decreasing access to healthcare lead to increased inequalities in healthcare utilization, adding to an already deteriorating health of this ageing population. Integration of on-site primary healthcare and OST could provide acceptable and accessible healthcare.

**Supplementary Information:**

The online version contains supplementary material available at 10.1186/s13011-022-00434-w.

## Introduction

Opioid use disorder (OUD) is a chronic relapsing condition with significantly increased mortality rates compared to gender- and age-matched general populations [1]. Even though entering OST decreases morbidity and mortality [2–8], the negative impact on health from direct, or indirect consequences of years of substance use takes its toll. Similar to the situation in other European countries Swedish OST patients constitutes an ageing OST population [9, 10]. This is both due to an increase of the average age of individuals in OST and an increase in age of individuals suffering from drug related deaths (DRDs) in EU (including UK, Norway, and Turkey). Many of those who have used opioids for a long time are now in their 40s and 50s [9, 10], presenting with a wide variety of somatic disease and a high prevalence of psychiatric comorbidity [11–13] adding to the complex needs within this heterogenous population. Significantly higher overall prevalence of multi-morbidity, higher disease severity [14, 15] and low degree of prevention and management of chronic diseases among OST patients, compared to matched controls [16]. Neoplasms, external causes, digestive, circulatory or respiratory diagnoses were found to be the most frequent reasons behind non-DRDs among Scottish OST-patients [17] indicating lack of access, or existence of barriers, to timely adequate treatment.

Although a growing body of evidence show a high prevalence of various somatic symptoms within the OST population [14–21], studies on general physical health, unmet healthcare needs and access to primary healthcare among patients in OST are sparse [21, 22]. Data from our previous study showed that OST patients, to a large extent, refrained from healthcare seeking even though presenting with a high prevalence of self-rated physical illness [18].

Barriers to healthcare seeking among people who use drugs (PWUD) have previously been identified as experience of dehumanization [23] and stigmatization [24–27] leading to avoidance or delaying healthcare seeking [23, 26] with a high risk of negative health consequences [25, 26]. Opioid related morbidity and mortality continuously present a worldwide public health crisis as the number of opioid overdoses continue to increase in several countries [28]. Even though a slight decrease in Sweden’s DRDs was shown in the years 2018 and 2019, EMCDDA reported Sweden to have the highest numbers of DRDs in EU, including UK, Norway, and Turkey [29]. While most of these fatalities involve opioids [30] access to OST is unequally distributed on a national level [31, 32], even though policy changes during the last decade have led to increased availability. The “zero vision”, meaning a drug-free society, is applied in all Swedish narcotic policies [33] leading to continuously restrictive policies and a limited provision harm reduction services [34]. Even in Sweden, where primary healthcare is tax financed and covered by the universal health insurance, structural discrimination and socioeconomic disadvantage poses barriers to healthcare seeking [35], correlates with unmet healthcare needs [36, 37] and incident and fatal opioid overdose [38].

Facilitators to healthcare seeking among PWUD and OST patients has previously shown that intervention models providing integrated, multidisciplinary healthcare [9] by targeted [39, 40], linked [41–43], referral or on-site [44–46] PHC in various degrees facilitate healthcare seeking.

This study aims to address a gap in literature by clarifying what contributes to this sub-optimal healthcare seeking among OST-patients, by identifying barriers towards, and factors able to facilitate, healthcare seeking. Increased knowledge and awareness among healthcare professionals and policymakers is essential for addressing stigmatization of people who use opioids (PWUO), to close the gap between provider and patient, and to prepare for a shift in service provision of a growing and ageing OST population.

By employing mixed methods, we aim to achieve more comprehensive understanding of barriers and facilitators experienced by patients in OST towards healthcare seeking. Derived from the framework of “risk environment” previous research on stigma regarding experiences of healthcare among people who inject drugs, was analyzed through micro-, meso- and macro-level stigma [26]. Analyzing the effects of stigma on health, we decided to apply a similar strategy, based on Stigma theory [47], whereas the main principles behind the theoretical framework on access [48] was used throughout the analysis of the barriers hindering access to healthcare and the facilitators which could be used to override these barriers.

## Methods

### Theoretical framework

During the initial analysis of the quantitative part, it soon became evident that stigma permeates all levels of society and diminishes individual abilities and possibilities in utilizing healthcare, which is why stigma theory, originated from the works of Goffman [47], was applied throughout the mixed methods analysis. Penchansky and Thomas [48] framework on access to healthcare, focusing on barriers to healthcare utilization, was originally chosen as the theoretical ground for analyzing the result as this model highlights the degree of fit between services provided and ability of the individuals to utilize these services.

#### Stigma

Goffman initiate the theory of stigma by describing the spoiled identity as the bearer of socially discrediting attributes which could exists as physical deformities, discreditable stigmas, hidden stigmas like addiction or mental illness, thought of as blemishes of the individual character, or tribal stigma due to race, nation and religion [47]. Categorization of individuals are however unavoidable as they are an integrated part of norms within structures of culture, generally not harmful. Thus, when categorization of negative stereotypes is endorsed in order to discriminate against individuals or groups, it can have tremendous negative consequences on their opportunities in life [25, 49].

For stigma to exist, differences between individuals are distinguished and labeled, where the individuals being labeled are linked to undesirable characteristics (negative stereotypes) defined by dominant cultural beliefs. Labeled individuals “them” are separated from “us” through oversimplifying traits or beliefs, placing “them” in distinct categories, where discrimination and status loss lead to unequal treatment and outcomes. However, stigma cannot exist if there is equality of power. Through unequal distribution of access of political, economic, and social power distinctions are made by us and them, and those not fitting in to accepted norms are disapproved of, discriminated of and excluded [49].

Our data primarily concern micro-level stigma; however, individual experience of stigma and self-stigma is mainly preconditioned by structural sanctions of macro-level stigma, created by the norms and rules in society, produced and reproduced on socioeconomic and political levels through policies and laws. These norms and rules are accepted by the public and reinforced by media, and permeated through institutions, such as the healthcare system and criminal justice system, through policies and laws [26]. It is through these structures that inequality of healthcare is shaped, in the absence of policies supporting marginalized groups and where criminalization of drug use creates the means which enables and impedes stigma on a structural level [50].

Meso-level stigma is macro-level stigma enacted by, and through, the encounters and interactions with, healthcare professionals (e.g., physicians, nurses, assistant nurses) [26]. Barriers to service provision and the inability, or failure, to meet the needs of individuals or groups leads to inequalities both in services and outcomes.

Micro-level stigma occurs on an individual level, such as prior experiences of being treated badly due to current or prior substance use. Self-stigma is the acceptance and adoption of constructed negative societal norms and beliefs of deviance and the internalization thereof. The process of self-stigma starts with awareness and agreement of stigma, where internalization thereof leads to negative impacts on self-esteem and self-efficacy, feelings of shame and not being worthy, often resulting in resignation, described as the “why try” effect [51, 52].

#### Dimensions of access

A moderated model of Penchansky & Thomas (1981) [48] describing dimensions of access is used in order in the analysis of the result in order to display barriers and facilitators in the dimensions of availability, accessibility and acceptability. *Availability* (including accommodation) represent supply and demand, volume and type of existing services and resources (provider) in relation to volume and need (patient). Organization of resources (hours of operation, appointment systems, drop-in and telephone services) in relation to acceptance by and ability of patients seeking healthcare and their perception of the appropriateness of these services. *Accessibility* (including affordability) representing the spatial factors in healthcare, location of services in relation to patient location (convenient location of healthcare, transportation, distance, travel time, cost). Patient ability not only to pay, thus including the perception of worth relative to total cost. *Acceptability* represents patients’ attitudes of characteristics of providers, both on a personal level (medical personnel and their attitudes in relation to personal characteristics of clients) and on behalf of the practice itself (intern and extern environment).

### Study design

To identify barriers towards healthcare seeking and to achieve a greater understanding of the different perspectives of what contributes to sub-optimal healthcare seeking among OST patients, we conducted a mixed methods study where the quantitative part consisted of cross-sectional questionnaire data, including both closed- and open-ended questions [18]. Our previous study presented data from the first part of the questionnaire examining self-reported symptoms among participants and whether they had sought healthcare for these symptoms. As the results showed a high prevalence of self-reported symptoms and a low prevalence of healthcare seeking the aim for the current study was to examine the reasons behind this low prevalence of self-reported healthcare utilization.

Using exploratory design, this study consists of two phases where the initial analysis of quantitative data gave rise to the need of further exploration into certain areas for a deeper understanding of the material. An equal weight design, (QUAN➔QUAL) [53], was created with two separate datasets. The results from the quantitative part of the study were built on the questionnaire [18], section Q28–29 (see Additional file 1), whereas the qualitative part consisted of semi structured interviews [54].

Mixed method design allows for the integration of both qualitative and quantitative data as methods triangulation fortifies and enriches study’s conclusion. The design is complementarily, as additional levels of perspectives offer a fuller understanding, adding another layer to the research question [53]. The goal was to establish an interview environment, where participant knowledge and experience are in focus and where the interviewer was open to new perspectives and insights, aiming to establish trustworthiness [55–57]. The principals of thematic analysis were employed to analyze and interpret the different aspects of the qualitative material [58].

### Setting

With its 350,000 inhabitants, Malmö is the third largest city in Sweden. At the time of the study there were five OST clinics in Malmö with 520 registered patients in total (October 2017, reported by representatives from each unit to first author KT). The questionnaire data was collected from four out of the five OSTs; three of which are part of public healthcare run by Addiction Center Malmö, and one (OST INM) providing private care, however tax financed and covered by the Swedish universal health insurance. OST is provided at specialized psychiatric treatment facilities, obliged (in Skåne Region, South Sweden) to provide not only pharmacological treatment with methadone or buprenorphine, but also a comprehensive psychiatric care, psychological and psychosocial treatment, regular testing for infectious diseases, and to conduct simpler somatic healthcare.

At the time of the study, two of the clinics provided on-site PHC for their patients, whereas the other two were about to implement on-site PHC. On-site PHC primarily targets OST patients without established PHC contact, or patients with non-sufficient PHC contact. If needed, OST staff motivate patients to book an appointment, keep track of the date and time of appointment and reminds the patient about the appointment. Patients with unstable housing or having other difficulties keeping track of appointments can receive help to arrange for their scheduled appointments to be sent to the clinic. If requested by the patient, OST staff will inform the patient about planned examinations and accompany them during the appointment with the on-site PHC physician. OST-nurses will assist the PHC physician in taking blood samples, blood-pressure, weight, saving the patient from visiting several places, trying to concentrate interventions to the OST unit which the patient is required to visit on regularly. Patients who are referred to specialized units for examination will, if required by patient, get information about the examination, how to prepare for this, where to go to and how to get there. Secrecy between the OST and the PHC clinic is standard unless the patient would like to arrange things differently.

### Quantitative methods

#### Data collection

For the quantitative part of the study participants were recruited at four different OST-clinics in Malmö, one private and three public facilities, through convenience sampling by OST staff or by the first author (KT), from May 2017 to March 2018, as described in detail previously in Troberg et al. [18]. Inclusion criteria for participation in the quantitative part of the study met the following criteria: 1) being registered as a patient in one of the four OST clinics, which also meant being prescribed methadone or buprenorphine, and 2) being 20 years or older (by default, unless special circumstances call for exceptions to be made) or older [59]. Exclusion criteria were inability to provide informed consent due to Swedish language difficulties, intoxication, or psychiatric disability. Patients were approached and asked in person if they were willing to partake after receiving written and oral information about the study. Upon expressing willingness to take part, informed consent was signed by the patient. During this period most patients had been approached more than once and were asked if they were willing to take part of the study. Patients declining to take part, or patients not allegeable during the timeframe described above, due to prison sentence or other institutional care, were not registered. Inclusion came to an end after a period of which all patients who were asked if they were willing to partake, either replied that they had already taken part of the study or were declining to take part of the study.

After informed consent was obtained, participants answered a questionnaire. Staff helped if there were questions about the questionnaire and could offer help with reading the questions and writing responses, if required by the patient. Reading glasses were available. The questionnaire has previously been described in detail by Troberg et al. [18]. The section of questions which was used for this part of the study regarded questions on healthcare seeking, refraining from healthcare seeking and reasons for refraining, presented in Additional file 1 (Appendix Questions 28–29).

Closed- and open-ended questions and data concerning reasons for patients not seeking healthcare for their symptoms or problems regarding somatic health, was the objective for the quantitative part of this study. No economic compensation was provided for answering the questionnaire.

#### Data analysis

Two demographic variables were dichotomized prior to analyzation: “unstable housing” included the following responses: “transitional apartment”, “institution/family care placement”, “hotel”, “homeless” or “other”. “Public assistance” or “other” was recoded into “unstable income”.

In seven cases participants had answered the questionnaire twice. Only results from the first questionnaire were included in the results. One of the questionnaires was excluded as every second page was missing, leaving the participant without the possibility of replying to questions covering the studied section. Eight questionnaires were excluded as participants had not answered any questions within the section included in this study. This section is referred to as Appendix Section Q28–29 (See Additional file 1).

If data was missing for the yes/no question regarding worry about physical health, though the open-ended reply described specific symptoms, the variable was recoded to “yes”. If replying “no” to the same question, replies which could only be given if the answer had been “yes” were regarded as missing. When yes/no questions concerning refraining from healthcare seeking were missing, or if the answer was “no”, thus following answers described reasons for refraining from healthcare seeking, the answer was recoded as “yes”.

Participants leaving comments not answering the question were saved, as they were written, in the variable “comments”, however, the variable was regarded as “missing data”. This was also the case if replies were not possible to read, or to be interpreted. Missing data were excluded from analyses.

Variables containing multiple choice questions in the studied section of the questionnaire were recoded into three new dichotomized variables. If responding “Find roof over my head”, “Getting money” or “Getting drugs” or describing “other reason” in regard to the main question concerning deprioritizing of health, this was recoded to the variable “Deprioritizing”. The question beginning with “It would not have mattered…” followed by multiple choice replies “Do not have money for medication, if prescribed”, “I would not have been able to follow doctors’ recommendations” or “Other reason”, was recoded into “Resignation”. The response starting off with “Called but…” followed by multiple choice replies “There was a telephone queue, and I did not have the patience to wait”, “There were no available appointments”, “I got an appointment, but missed it” or “Other reason” were recoded to “Tried without success” (See Additional file 1).

The results from the questionnaires were registered in SPSS version 21.0 (IBM Corp. 2012) for descriptive analyses.

### Qualitative methods

#### Data collection

Interview data were collected from two of the public OST clinics using semi-structured interview containing both close-ended and open-ended questions [54, 55]. Study participants for the interviews were recruited between February 2018 and March 2018. A sub-sample of the questionnaire participants, who had experienced unmet healthcare needs, were invited to participate. The study was also advertised through flyers posted at each site, and clients interested in partaking could either contact the interviewer or staff at their clinic. Convenience, strategic sampling was used by one of the authors (KL) who approached potential participants in the OST clinics’ waiting areas. Participants received oral and written information about the study and signed a written consent before participation. Interview participants received a grocery store gift voucher valid for SEK 100 (≈ 10 USD). Twelve interviews were conducted, one was excluded due to participation inclusion criteria not being fulfilled. A total number of 52 patients were approached but 40 declined, mainly due to time limitation or lack of interest.

Exclusion criteria were inability to provide informed consent due to Swedish language difficulties, intoxication, or psychiatric disability.

All interviews, approximately 30 min long, were conducted by the same author (KL). The questions included sections covering three head subjects, with the possibility to adding qualifying follow up questions. The interviews concerned questions about the experience of health care, lifestyle, and self-images and finally their expectations and ideals concerning Swedish health care and what the interviewees believed would facilitate their own healthcare seeking. The interviews were recorded with a digital voice recorder and transcribed by the interviewer.

#### Data analysis

In relation to trustworthiness which refers to credibility, dependability, confirmability, reflexivity, and transferability [55–57]*. Credibility* (internal validity) may be enhanced by prolonged engagement, triangulation, peer debriefing and researcher credibility. This was thought to be fulfilled both as participants at any time could question or comment interpretation of their answers to the questions and by the prolonged engagement as members of the research group have many years of experience of working clinically within the field and of research within the field and at OST clinics where interviews took place. *Dependability* (reliability/data stability over time and conditions) was sought through initially testing the interview in relation to content of themes and questions, and length on interview, by a representative from the Drug Users Union, Skåne.

The interviewer strived not to ask the participants leading questions or interrupt while speaking. In relation to the exploratory design the interviewer had an openminded approach with the aim of gaining new insights and perspectives in relation to interviewees experiences. The pilot interview was analyzed and discussed through peer debriefing within the research group and with the representative from the Drug Users Union, before including and interviewing individuals in the study. For continuity, all interviews were performed by the same researcher (KL), who also transcribed the material. *Dependability* was also obtained through separate full analysis of the interviews by two of the authors (KL and KT), individually listening to the recorded material and repeatedly going through the transcripts according to thematic analysis process [58].

Thematic analysis approach was used to interpret the different aspects of the material [58]. The transcripts were reviewed thoroughly, through repeated reading, to get fully acquainted with the material before seeking to describe inherent patterns, generating initial codes. During this process ideas on possible patterns and meanings were noted. In a first attempt to organize data into meaningful groups, codes, which were gathered under potential themes, including all data which was relevant to each of these. Mapping out each theme, sub-theme, and its content, relations between codes and themes were reviewed, certifying that each coded extract was significant not only in relation to the theme, but also its validity to the full data set, before finally defining and naming themes.

After individually analyzing the material, results from each analysis were revised by the third author (DD). Any discrepancy between the two analyses were discussed, negotiated, and re-analyzed within the research group, aiming to reach *confirmability* (objectivity/neutrality of the data). Considerations on acknowledging researchers’ *reflexivity* and its influence on the research process were discussed and taken into the process. The context in which the interviews are preformed is likely to present varieties if they were to be performed in a completely different settings, although main findings are most likely to meet the debated criteria of *transferability* (generalizability).

Fictitious names were used to protect the confidentiality of the participants.

## Results

### Quantitative part

#### Description of questionnaire sample

Participants who had answered any of the questions in the section of the questionnaire concerning reasons for refraining from healthcare seeking (See Additional file 1) had a mean age of 43.8 years, 28% were female, 76% were born in Sweden, and 21% reported having unstable housing. Unstable income was reported by 59, and 75% were daily smokers (see Table 1). These numbers were representative compared to respondents (*N* = 218) who had answered the entire questionnaire in full, or to a high degree [18].

**Table 1:** Characteristics of study participants (*n* = 210)

	n (%)
Age (mean; SD)	43.8 years; 10.1
Age (median; range)	43 years (23–67)
Female	59 (28.1)
Born in Sweden	160 (76.2) ^a^
Unstable housing	44 (21.0) ^b^
Unstable income	121 (59.0) ^c^
Daily tobacco smoking	150 (74.8) ^d^

^a^ missing *n* = 1 ^b^ missing *n* = 4 ^c^ missing *n* = 5 ^d^ missing *n* = 8

Missing values excluded from denominator

#### Reasons for not seeking healthcare when needed

Reportedly, the most common reasons for not seeking healthcare when needed was due to deprioritizing (49%), fear of being labelled as a junky and not getting helped (47%) and fear of being treated badly (38%). Almost a quarter (23%) had tried to get an appointment, but claimed that they had not succeeded, whereas 20% reported giving up before even trying, stating that whey would not be able to follow through with a presumed treatment anyhow. Avoidance of healthcare seeking due to being afraid of there being something seriously wrong with them was reported by 17%. Twelve percent reported being worried that they might not understand, as the reason for not seeking healthcare and between four and 9 % replied not knowing which primary healthcare unit they were listed at, what number to call, or could not make the call due to lack of phone or money to call for (See Table 2).

**Table 2:** Reasons for not seeking somatic healthcare when needed (*n* = 109) (Multiple answers possible)

	n (%)
Deprioritizing	53 (48.6)
Fear of being labelled a junky and not getting helped	51 (46.8)
Afraid of being treated badly	41 (37.6)
Tried without success	25 (22.9)
Resignation	22 (20.2)
Worried about being seriously ill	19 (17.4)
Worried that I would not understand	13 (12)
I did not know what number to call	10 (9.3)
I did not know I was listed (or where to call)	9 (8.3)
I did not have a phone (or money to call for)	4 (3.8)

### Qualitative part

#### Description of interview sample

The sample were predominantly in their middle age (median 43 years, range 26–61 years). Eight participants were men, and three responders were born outside Sweden. The analyses of the eleven interviews generated four themes connected to barriers, and one connected to facilitators. Collectively 16 sub-categories were found to capture the contents of the themes connected to barriers. Six sub-categories were found to represent the theme of facilitators, which are presented in Table 3.

**Table 3:** Healthcare utilization - Barriers and facilitators

**Barriers**	
Substance use disorder ➢ Functionality ➢ Cognition ➢ (De-)prioritization ➢ Self-medication	
Fear of stigma ➢ Discrimination ➢ Humiliation ➢ Mistrust ➢ Judged/Labelled ➢ Unprofessional behavior medical staff: focusing on drug use instead of problem presented. Not listening/taking patients seriously. Lack of knowledge and understanding.	
Self-stigma ➢ Shame ➢ Blame (oneself) ➢ Not being worthy ➢ Situation caused by oneself	
Difficulties navigating throughout the Healthcare System ➢ Not knowing where to go/whom to contact ➢ System not cohesive ➢ Lack of information ➢ Difficulties keeping appointments	
**Facilitators**	
Cohesive healthcare ➢ On-site ➢ Convenience - Going there anyway (to OST clinic) ➢ Support incl. Booking appointment/ reminding ➢ Trust - Safe space ➢ Being listened to /taken seriously. Being someone who matters ➢ Dedicated, professional staff	

#### Substance use disorder

Relapse, or longer periods where additional drugs were used continuously, was often described by participants as periods where other areas in life would be deprioritized. The compulsion to find drugs and money to support an addiction preceded practically all other needs. All but one of the interviewees expressed deep concerns and worries about their health. However, these needs, and concerns were not prioritized during relapse, as they were forgotten, ignored or pushed back, as described by Paul, a man in his early 60s:

*You forget. You simply don’t think about it at all. There are plenty of other things that needs to be taken care of, and time just flies. Seeking healthcare is not something you prioritize while actively using drugs*.

Jane, a woman in her 40s, described further how the consumption of benzodiazepines affected her:

*I could have done more than I did, but it’s not that simple. I started consuming a lot of benzos, which resulted in everything in life spiraling out of control. It also leads to the inability for me to feel or locate pain or feeling hungry or tired. You just go numb while consuming pills.*

Combined with other barriers and issues that seem more pressing at the time, the importance of seeking healthcare seem to fade.

#### Fear of stigma and unprofessional treatment as a barrier

The large proportion of questionnaire respondents claiming fear of stigma and of being treated badly as the main reason for not seeking healthcare was confirmed by the majority of the interviewees. Previous experience of stigma was reported to be one of the main reasons behind avoiding healthcare seeking. Adam, a man in his mid-20s described what it could be like in a waiting room, enduring being stared at and judged, and at the same time anticipating that the only thing that was most likely to result from this tournament was being labeled, mistrusted, and then getting a pat on the back and sent back home:

*Believing that hospitals and primary healthcare centers should be places where one would not be judged. People are ill, and addiction should be considered as an illness, but it’s not like that. [When] You go there and… people are looking at you in a certain way. You are being judged as an addict and it is hard to sit there, in the waiting area, when people are looking and judging, especially when, in the end, you don’t get any treatment at all. If you seek help when you are in pain, then they will only think that you are trying to score some drugs.*

The mistrust described above by Adam, where medical personnel suspect patients to be “fishing” for prescription drugs being the reason for seeking healthcare. When Paul had been rushed to the emergency department with hematemesis, a few weeks ago, the first thing Paul was asked by the physician entering the room was: *“What prescription do you want old man?”.* Mistrust was highlighted by many of the interviewees, especially among those suffering from long-term pain. Not receiving any help, patients felt that they did not have any other choice than to find a solution elsewhere, which often included buying drugs illegally.

Participants also expressed that there had been moments when they had been treated like “everyone else”, but as soon as information reached the medical personnel that the patient was suffering from addiction, or was a patient at the OST clinic, things changed, as described by Peter, a man in his late 40s:

*[You are] being treated poorly. Worse treatment and less accurately diagnosed. Put simply, a less warm reception. As soon as it comes up that you are a drug addict you will be treated differently. That’s a fact*.

Sarah, who had just given birth, experienced being treated badly after it came to the medical staffs’ attention that she suffered from opioid addiction. This led to her deciding to leave the hospital early, against the recommendations by the obstetrician, stating that after this experience she would wait until being almost dead before ever seeking healthcare again.

Previous experiences of stigma within the healthcare system were described by the majority as the reason why they felt forced to avoid healthcare seeking until the very last minute, until there was no other option than to seek acute care. Amanda, a woman in her late 30s, was brought to the emergency room by a friend, worried about her health:

*Well, it’s just that I don’t call. Finally…one time I didn’t call and didn’t go to the doctor, nothing, which ended with someone taking me to the hospital. When I fell over the doorstep, my breathing was 20%*.

Interviewees shared multiple stories on how the consequences of healthcare seeking avoidance had led to increased pain and suffering on a daily basis.

#### Self-stigma and internalization as a barrier

Interviewees not only described discrimination and stigma, but also how these encounters had confirmed the negative feelings they already had about themselves. Darren, a man in his late 20s, described how hard it was when he realized that he had become *“one of those whom parents warn their children about”*. This realization made him feel shame and failure as he blamed himself for the situation he had ended up in:

*That’s why it feels so hard. Because it is a failure in a way. That failure is not something you would like to tell everyone about, or show anyone, and I think that is a large part of the reason why you don’t seek medical help, because you are ashamed. In some way this is something you have created yourself, through your destructive way of living.*

Darren later concluded that the shame and guilt emerging from the situation when medical staff found out that he was suffering from addiction, was what made him avoid seeking healthcare, and what had made him avoid it in the past*.* Amongst other interviewees, Jane also referred to shame and guilt as reasons for not seeking healthcare, adding that she felt that there also was gender aspect to shame. Being a woman addicted to heroin, was thought of as being even further from the social norm:

*I do think that we as women somehow are even more ashamed. Being addicted. It doesn’t fit the picture of someone being addicted to drugs or heroin, or someone in the methadone program. Being a woman and a mother and all that.*

Being treated poorly was considered to confirm their own beliefs of being outcasts, and the notion that they should in fact be ashamed of themselves, for making choices in life that had left them in this situation. One of the interviewees even stated that he felt that he, because of this, was not even worthy of seeking help. He also described that not only did previous experiences of stigma, but the pure anticipation of being stigmatized, make him interpret situations as discriminating, which may, or may not, have been the case:

*It’s just that being ashamed about your situation, sometimes makes you think that there is more to it than it actually is. Maybe she [medical staff] thought or did something in a way or another, but I interpreted it differently because of the fact that I’m so ashamed that I directly connect her action being about my addiction.*

#### Difficulties navigating throughout the healthcare system as a barrier

A few interviewees described that lack of knowledge on when, where, and how to seek healthcare stopped them from even trying, which was also found among responses in the questionnaire. In addition, not being able to keep appointment was perceived as yet another barrier, as missing an appointment let them to feeling even less worthy of getting another chance. Several participants stated that even though it seemed simple at first it all became too much to keep track of later down the road. For Jane and others who visited the OST clinic daily, expressed that the procedure of referrals as too much of a hassle, being unable to handle multiple contacts with healthcare. It was enough just going to the clinic:

*One does not always know where to turn to either. Most often there’s the process where a referral has to be arranged, you have to leave blood samples and then you have to wait for the results and maybe you have to phone at certain hours. If you don’t make an appointment and all that, you tend to drag the whole thing. You know, you have to call between certain hours. It is a lot to keep track on and at the same time I have to run up here [OST clinic], and then… it all gets a bit stressful.*

As confirmed by other participants, getting a referral after a visit to the primary healthcare clinic was perceived as complicated and stressful. Instead, as described by Adam, going directly to the ER, when the problem had gotten so bad that there was no other option, was often looked upon as a more rational choice.

*You get to leave some blood samples and then they will give you a new referral to somewhere, and then you’ll have to have to go there for two or three visits in order to finally get to the place you really wanted to be referred to in the first time. That’s why it sort of feels like I might as well just head straight to the emergency unit and get everything sorted there. They will check you, draw some blood, send you on a gastroscopic examination, or wherever, and then its goodbye.*

Difficulties in navigating throughout the healthcare system, or not being able to “fit into the system” left a great deal of patients on the “outside” where self-medication was often seen as the only option. Interviewees were however aware that this might lead to negative consequences, as described by Jane:

*Somehow it becomes easier. If I smoke some weed, then I won’t be in as much pain, or if i take a few pills, then I won’t notice it, and I will sleep better and all that. Sure, that’s the way it is, but on the other hand, this triggers my addiction. So no, it is not the best solution.*

#### Facilitators

As described by the majority of the participants, on-site PHC at the OST clinic seemed to increase the possibility for provision of a more comprehensive healthcare. Several patients expressed that they generally would like healthcare to be cohesive, as they felt it would increase their abilities to access healthcare.

Trust and acceptance by healthcare providers seemed to be crucial to the participants. Interviewees underlined that they wished medical personnel to focus on the reason for seeking healthcare, and not to focus on their use of illicit drugs. They wished to be taken seriously, being listened to and understood. Two of the respondents implied that increased knowledge and experience on behalf of medical staff would likely attribute to a more professional behavior, and less stigma, as expressed by Darren:

*It would be much easier if healthcare personnel would have experience and knowledge of drug use. I think that would attribute to the feeling of acceptance in some way.*

A few participants brought up the needle exchange program (NEP) as being a “good example” when it came to healthcare, a place where one can turn to “with everything”, not having to be subject to discriminating treatment. The majority of respondents also expressed satisfaction when it came to the services provided by their OST clinics and thought that they “did enough”. Two participants even expressed a sense of pride in being a patient at one of the OST facilities. Sarah, who described herself as an outsider when it came to other situations, expressed that the facility represented a “safe space” and that the staff were actually proud of her and of what she had accomplished:

*They are so proud of me, I know they are. The way that I have dealt with everything. The doctor even told me that I’d done very well for myself. Sometimes you just need to hear that*.

Patients described that as they were visiting their OST clinic regularly, in some cases daily, having access to PHC on-site was essential, as the need to visit more than one healthcare facility was perceived as stressful. One of the participants also mentioned unstable housing as a barrier and thought it would be easier for if they had access to on-site PHC. As OST staff previously had offered Amanda to administer her referrals and appointments, she expressed that easy access and support from OST staff had helped her to become more motivated to deal with issues concerning her physical health:

*I can book an appointment when I’m there, and they [OST personnel] remind me one day prior to my appointment. This has given me a push as well. This [on-site] has worked out very well for me*.

Several participants thought that the support of OST staff, when it came to bookings and reminders of appointments, was an important contributor to accessing somatic healthcare, even when it came to referrals to other units, as described by Adam:

*They [staff at OST clinic] helped me here and I thought it was great. I wouldn’t have dealt with it otherwise /…/ this makes me feel as If I actually will receive help for my problems*.

## Analysis & discussion

This mixed-methods study is, to the best of our knowledge the first study set out to examine barriers towards healthcare seeking among patients in OST. According to the result from the questionnaire, the most common reasons for not seeking somatic healthcare, in spite of a perceived need, were deprioritization, fear of stigma and of being treated badly. These results are similar to the ones of a survey in Albany, New York, investigating barriers to general healthcare among clients utilizing syringe services, where the most prevalent barrier was found to be deprioritization of medical care (trying to ignore problem and procrastinate), whereas judgement by clinicians was perceived to be the biggest barrier [60]. While almost half of the questionnaire respondents declared deprioritizing as one of the reasons for not seeking healthcare when needed, stating that there were issues that seemed more pressing in the situation they were currently in. Applying mixed methods allowed studying an additional dimension, showing that reasons for not seeking healthcare, such as deprioritizing, was deeply intertwined and hard to interpret only from quantitative results.

Interviewees added that during periods of relapse they did not really have the choice to focus on their health, as everything else, except drugs and getting money to buy drugs, seized to exist. However, this did not imply that issues concerning health was not of great concern, as more than half of the respondents to the questionnaire stated that they were worried or concerned about their physical health. The majority of interviewees described being worried about their physical health, as they were highly aware that the lifestyle surrounding an opioid addiction also implies an increased risk of a broad range of diseases. This was also reflected by a rather high proportion of individuals stating that they had not sought healthcare since they were worried about being seriously ill.

During the interviews it became clear that the term “deprioritizing” consisted of an array of contributing factors. Meso-level stigma was recurrently described medical concerns not taken seriously by medical personnel. Patients described this as not being listened to, being mistrusted, judged, labelled and treated badly. In many cases the interviewees stated that there was no point in even trying as acceptable healthcare was considered highly unobtainable. Mistrust, prior experiences of being treated badly added to difficulties in finding suitable treatment, may reflect why a majority the OST patients suffering from pain did not seek healthcare [18], as described by several of the interviewees.

Meso-level stigma towards patients with SUD among healthcare professionals is well documented and the likelihood of these patients will receive suboptimal treatment is thought to be high [61]. Diseases, such as opioid addiction, which are perceived to be controllable, have shown to cause a higher degree of negative judgements and attitudes towards these patients [62–64], with a risk of healthcare decisions to be based on moral, rather than medical, judgements.

Previous experiences of stigma in the healthcare setting clearly led to avoidance of healthcare seeking, depicted by the high proportion of the questionnaire respondents stating fear of stigma and of being treated badly as the reason for not seeking healthcare. This was also confirmed by the majority of the interviewees sharing their experiences of being treated badly because of their opioid use disorder, which caused avoidance, procrastination, trying to find alternative solutions, delaying healthcare seeking until there were no other way that to turn to the emergency department. Similar responses were found in a study on methadone maintenance treatment as social control, where Australian patients reported avoiding healthcare seeking due to own, or others, experiences of stigma and of their health problems not being taken seriously. This study also pointed to how micro-level internalized stigma was depicted by the participants, referring to themselves as “junkies”, or “the plague of society”, internalizing and perpetuating the structural stigma in society [27], also described among our interviewees. The shame of having caused this situation themselves made them reluctant to seek healthcare. The feeling of being unworthy of healthcare seeking, blaming themselves for the situation they had ended up in were recurrently described by our participants. Interviewees also reported continuously experiencing the same stigma only by being an OST patient, only taking medication according to prescription. On a similar note, macro-level stigma towards methadone was reported by interviewees in California, based on the public stigma equating methadone with illicit drug use [26], which also increases the risk of OST avoidance. Previous experiences of micro-level stigma are a strong driving factor behind label avoidance, thus not seeking healthcare when needed [51]. This was frequently reported both through respondents of the questionnaire and among our interviewees. Resignation, described by several participants, as no point in even trying, could partly be explained by the negative impact internalized stigma often has on self-esteem and self-efficacy, often expressed as hopelessness, resignation (“Why try”) [51, 52].

The dimension of gender and stigma was brought to attention by one of the interviewees, as she described that by being a woman, addicted to heroin, or in OST, was even further from the social norm of how women and mothers were supposed to be like. Research on stigma in relation to women and drug use show how especially using “hard drugs” such as heroin or crack cocaine violates social norms and expectations of womanhood [65]. Not only do women seem to face a higher degree of stigma during drug use, but also after quitting drug use [66], making it harder to escape the labels of addiction.

There was an additional aspect of difficulties as patients not only found it challenging to booking, but also keeping track on, appointments and navigating throughout the healthcare system itself. Spatial separation of specialized healthcare, and separation into different fields, made interviewees feel confused about where to turn to with their problems. Also attributed to resignation, answers from the questionnaire, stated that it would not have mattered if they had gotten an appointment as they would not be able to follow doctors’ instruction, and/or could not afford picking up prescribed medication. Quite a few had tried to get an appointment, but somehow failed, whereas a few did not even know where to turn to. As studies have shown, OST patients have a high degree of psychiatric comorbidity [11–13] and a low degree of health literacy [67] most likely leading to an array of negative effects on health, healthcare seeking [9] and engaging in one’s health, as the system also poses a high degree of responsibility on the individual. Here, providers have failed to offer available and accessible healthcare since the responsibility placed on the individual is unreachable for some. When it comes to navigation within the healthcare system Sofaer and colleagues claim that policymakers are responsible for the design of a system that actively reduces barriers, especially considering the various difficulties among vulnerable members of society [68]. On a macro-level, structural and systematic exclusion of vulnerable groups has negative effects on an array of life opportunities, including health [69].

From this standpoint deprioritizing and procrastination encompasses both subtle and profound barriers, and as long as presumptive negative outcomes overrides possible and presumptive positive outcomes, healthcare seeking will most likely be postponed, until there is no other solution than to seek emergency care. Not dealing with these barriers will inevitably increase suffering for the individual and render increased costs for society. The consequences for an ageing OST population [9] with a high burden of psychosocial challenges [11–13] and somatic disease [14–21] with sub-optimal access to healthcare, are an increased risk of extended suffering, self-medication, increased morbidity, prolonged treatment, in-patient care and increased use of emergency care [44, 70, 71].

Healthcare should be offered without negative attitudes and discrimination on behalf of medical personnel, as the basic principles of the public healthcare policy in Sweden is based on laws emphasizing equality and care on equal terms [72]. In the await of changes in the policies and law supporting a continued stigmatization of marginalized groups, the challenge of providing access to healthcare based on equality could only be achieved if these barriers will be dealt with in a way which will be *acceptable* to marginalized individuals. As suggested by one of the interviewees, medical staff should have sufficient knowledge about addiction, in order to offer patients a professional encounter. Studies have shown that one of the causes of meso-level stigma is due to deficiencies among healthcare staff in general when it comes to knowledge about substance use [61]. This led to uncertainties and anxieties among staff, fed by public stigma of people who use drugs as dangerous and manipulative.

While a large proportion of patients suffering from OUD manages to recover successfully, there is a high predisposition of relapse [1]. During these periods there is an increased risk of direct and indirect physical injuries [73], however, at the same time several patients are finding it hard to prioritize health indicates an increased need of *accessible* and *available* somatic healthcare. Added to this we also have an ageing OST population, where physical ageing, to a large extent, is accelerated by cumulative direct and indirect effects of years substance use, which will increase future need of age-specialized services with multidisciplinary care [9], there is clearly a high level of healthcare needs within this population. Increased PHC availability and accessibility would mean providing services located to where the patients are and in relation to the needs of the patients *(availability*). Organizing the appointment system, including drop-in and telephone services, according to needs and abilities of our patients (*accessibility*) would facilitate healthcare seeking. If patients are not able to seek help through the existing system, then the system has to change.

For individuals with prior experience of stigma and discrimination, the chances of them seeking healthcare when needed is most likely increased if they were offered *acceptable* healthcare. When interviewees referred to staff at NEP they mentioned “trust” as a prerequisite for “good” [acceptable] healthcare, not being punished for their substance use, being cared for and treated “just like any other member of society”.

Integration of on-site PHC could contribute to increased motivation and utilization of healthcare and decrease use of emergency departments and inpatient care [44, 70, 71], without an increase in cost [44]. Also, by the reflections from participants of this study, it seems to increase *availability* and *accessibility* by offering *acceptable* healthcare in a supportive and familiar environment.

This study suggests that employing multidisciplinary teams, with well-established contact within the patient group, could, by working together to meet the needs of OST patients with previous negative experiences of healthcare and with difficulties in navigating throughout the system, override at a large proportion of the barriers which hinders patients to seek and receive healthcare. As this study has its limitations there is a need for future research explore general somatic health within the OST population, assessment of on-site PHC and multidisciplinary targeted intervention, and assessment of resources and stigma among healthcare personnel.

This study is subject to several limitations. Depending on self-reported data could present limitations, thus, to capture patients’ experiences, self-reported data is the best method [74, 75]. Representativity of the quantitative part of the study has previously been described in Troberg et al. [18].

As for the qualitative part of the data, there is always a risk that patients declining participation could be patients who carry a heavier weight of unmet healthcare needs and are more vulnerable. The sample was representative in relation to age and gender among registered patients at the four units where the study took place [18] and interviews showed a rich variety of information and inclusion of patients continued until reaching information saturation, where no new perspectives were presented within covered topics. As with all self-reported data, there is a risk of recall bias as participants were asked to describe situations and experiences that took place some time ago, however, these are the experiences and memories which participants carry with them. Before and during the construction of the questionnaire, and throughout the analysis process, the principles of trustworthiness was thought of and discussed in among the colleagues working with this study. As the first author of this paper was at the time also working clinically as a nurse and head of one of the units, it was decided that KT was not to conduct the interviews, as it could influence patients’ answers and could affect objectivity and would probably have a negative impact on conformability.

## Conclusions

The study results suggest that barriers surrounding the healthcare system leads to a high degree of unmet healthcare needs in an ageing population with a heavy burden of physical symptoms. Data from the questionnaire showed that deprioritization, fear of stigma and of being treated badly was found to be the most common reasons for not seeking healthcare, which was confirmed by the interviewees. However, deprioritization (and resignation) seemed to be sum of anticipation of negative outcomes, related to previous experiences of stigma and of being treated badly, individual means to navigate throughout the system, possibilities to follow instructions or prescriptions, in relation to possible positive outcomes. Deprioritization of one’s health was also greatly affected by periods of relapse, having no means to prioritize health. Avoidance, procrastination, and self-medication was often seen as better solutions as access to acceptable healthcare often seemed unavailable and unobtainable. Seeking emergency healthcare was often the last option when there was no other solution. Current structures of healthcare seem to contribute to increased inequalities, and further deterioration of health. The mere anticipation of a stigmatized encounter with healthcare, added to internalization of stigma, caused patients to avoid seeking medically motivated healthcare. By addressing a gap in literature on barriers and facilitators in healthcare seeking among OST patients, this knowledge can raise awareness among healthcare professionals and policymakers which is essential for addressing stigmatization of PWUO, decreasing the gap between provider and patient, and preparing for a shift in service provision of a growing and ageing OST population. On-site primary healthcare could be one way forward, integrating primary healthcare and OST, creating opportunities for acceptable, available, and accessible healthcare. Further investigations into somatic healthcare and healthcare utilization within this population is essential to create awareness and to fight stigma and inequalities.

## Supplementary Information

**Additional file 1: Appendix Section Q28–29.** Questionnaire section regarding healthcare seeking, access, and encounters with medical staff.

## Data Availability

The data used to support the findings of this study are restricted by the Regional Ethics Board, Lund, Sweden, to protect patient privacy. Data are available from the corresponding author on request, for researchers who meet the criteria for access to confidential data.

## Abbreviations

DRDs: Drug related deaths
NEP: Needle exchange program
OUD: Opioid use disorder
OST: Opioid substitution treatment
PHC: Primary healthcare
PWUD: People who use drugs
PWOU: People who use opioids
