# Supplementary material for: Healthcare seeking among Swedish patients in opioid substitution treatment – a mixed methods study on barriers and facilitators
Source: Subst Abuse Treat Prev Policy. 2022 Feb 5;17:8. doi: 10.1186/s13011-022-00434-w (PMC8817477; doi:10.1186/s13011-022-00434-w)
Supplement: Supplementary file 1 — Additional file 1: Appendix Section Q28–29. Questionnaire section regarding healthcare seeking, access, and encounters with medical staff. [file 13011_2022_434_MOESM1_ESM.pdf]

Appendix Section Q28-29
Healthcare seeking, access and encounters with medical staff

28. Are you worried about, or troubled by, a matter concerning your physical health?

O No

O Yes if =Yes → 28a. What are you worried about or troubled by?

____

28b. Have you sought healthcare for this matter?

O Yes → What kind of healthcare?

____

O No → Why not?

____

29. During the last year, did you ever refrain from healthcare seeking in spite of experiencing a need to do so?

O No

O Yes if =Yes →

29a. Why did you refrain from healthcare seeing in spite of a need to do so?

Recoded variable: ”Deprioritizing”

- ☐ I did not prioritize the need as it, at the time, was more important to
  - Find roof over my head
  - Getting money
  - Getting drugs
  - Other reason:_________________________
- ☐ I was afraid to find out that I had a serious condition
- ☐ I was afraid of being treated badly
- ☐ Fear of being labelled a junky and not getting helped
- ☐ I was afraid not to understand

Recoded variable: ”Resignation”

- ☐ It would not have mattered if I sought healthcare as I would not have been able to fulfil any treatment anyway as I…
  - Do not have money for medication, if prescribed
  - I would not have been able to follow doctors’ recommendations
  - Other reason:__________________________

- ☐ I did not know what number to call
- ☐ I did not know which primary care facility I was listed at (where to call)
- ☐ I did not have a phone (or money to call for)
- ☐ I called but…
  - There was a telephone queue and I did not have the patience to wait
  - There were no available appointments
  - I got an appointment, but missed it
  - Other reason:________________________

Recoded variable: ”Tried without success”
